# High-resolution NMR structures of the domains of *Saccharomyces cerevisiae* Tho1

**DOI:** 10.1107/S2053230X16007597

**Published:** 2016-05-23

**Authors:** Julian O. B. Jacobsen, Mark D. Allen, Stefan M. V. Freund, Mark Bycroft

**Affiliations:** a MRC Laboratory of Molecular Biology, Hills Road, Cambridge CB2 0QH, England

**Keywords:** SAP, Tho1, RNA

## Abstract

THO is a multi-protein complex involved in the formation of messenger ribonuclear particles (mRNPs) by coupling transcription with mRNA processing and export. THO is thought to be formed from five subunits, Tho2p, Hpr1p, Tex1p, Mft1p and Thp2p, and recent work has determined a low-resolution structure of the complex [Poulsen *et al.* (2014 ▸), *PLoS One*, **9**, e103470]. A number of additional proteins are thought to be involved in the formation of mRNP in yeast, including Tho1, which has been shown to bind RNA *in vitro* and is recruited to actively transcribed chromatin *in vivo* in a THO-complex and RNA-dependent manner. Tho1 is known to contain a SAP domain at the N-terminus, but the ability to suppress the expression defects of the *hpr1*Δ mutant of THO was shown to reside in the RNA-binding C-terminal region. In this study, high-resolution structures of both the N-terminal DNA-binding SAP domain and C-terminal RNA-binding domain have been determined.

## Abstract 2

In this study, high-resolution structures of both the N-terminal DNA-binding SAP domain and the C-terminal RNA-binding domain of *S. cerevisiae* Tho1 have been determined.

## 1. Biological context   

The delivery of translationally effective ribonuclear particles (mRNPs) to the cytosol is a complex process in eukaryotes that requires the integration of numerous processes including transcription and processing of pre-mRNA, formation of mRNPs and export from the nucleus (Köhler & Hurt, 2007 ▸). A vast array of proteins are involved in these processes and their interactions are carefully controlled to facilitate the delivery of mRNPs to the nuclear pore complex. Loss of control at any point can result in cellular mechanisms degrading mRNPs before they are exported (Houseley *et al.*, 2006 ▸).

An essential component of early mRNA biogenesis is the THO complex, which in the yeast *Saccharomyces cerevisiae* is composed of Tho2p, Hpr1p, Tex1p, Mft1p and Thp2p. The exact mechanism by which it function is unknown, but it is thought to bind RNA polymerase II during transcription *via* its polyphosphorylated C-terminal domain (Meinel *et al.*, 2013 ▸). THO has also been shown to bind Yra1p and Sub2p to form a complex known as TREX (Strässer *et al.*, 2002 ▸). The THO complex also mediates interactions with several additional proteins to stimulate co-transcriptional recruitment to nascent mRNA transcripts (Hurt *et al.*, 2004 ▸; Zenklusen *et al.*, 2002 ▸). Depletion and/or knockout of individual THO complex components *in vivo* has revealed that THO is not only involved in mRNA biogenesis but also takes part in preserving genome integrity (Aguilera, 2005 ▸; Huertas & Aguilera, 2003 ▸).

Tho1 was identified as a multicopy suppressor of *hpr1*Δ (Jimeno *et al.*, 2002 ▸; Piruat & Aguilera, 1998 ▸) and was thought to function in a similar manner to the yeast protein Sub2. Studies revealed that Tho1, like Sub2, can assemble onto the nascent mRNA during transcription and that Tho1 and Sub2 can provide alternative pathways for mRNP biogenesis in the absence of a functional THO complex (Jimeno *et al.*, 2006 ▸). Null mutants of *THO1* did not result in a distinct phenotype and thus the function of Tho1 *in vivo* remains unclear. However, the ability of Tho1 to suppress *hpr1*Δ was shown to be located in the RNA-binding C-terminal region. Our study has determined the solution structures of both the N-terminal SAP domain, which in other proteins has been shown to bind to DNA (Göhring *et al.*, 1997 ▸), and the C-terminal domain thought to be responsible for RNA binding. The SAP domain contains a helix–extended-loop–helix motif similar to those found in other members of this family and binds to DNA. The C-terminal region adopts a helical fold similar to that of the WHEP RNA-binding domains of metazoan aminoacyl-tRNA synthetases (Cahuzac *et al.*, 2000 ▸).

## 2. Methods and experiments   

### 2.1. Domain architecture of Tho1   

The domain architecture of yeast Tho1 was analyzed using *JPred* (Cuff *et al.*, 1998 ▸) and *Phyre* (Kelley & Sternberg, 2009 ▸) to identify regions that are likely to have a discrete fold.

### 2.2. Expression and purification of Tho1 N-terminal and C-terminal domains   

DNA encoding the N- and C-terminal domains of Tho1 were amplified from *S. cerevisiae* genomic DNA by PCR and cloned into a modified pRSETA (Invitrogen) expression vector that produces proteins fused to the N-terminally His_6_-tagged lipoyl domain of *Bacillus stearothermophilus* dihydro­lipoamide acetyltransferase. The resulting plasmids were transformed into *Escherichia coli* C41 (DE3) cells. Cells were grown in 2×TY medium at 37°C to mid-log phase and were induced with 1 m*M* IPTG. The temperature was reduced to 22°C and the cells were grown for a further 16 h. Isotopically labelled domains were prepared by growing cells in K-MOPS (Neidhardt *et al.*, 1974 ▸) minimal medium containing ^15^NH_4_Cl and/or [^13^C]-glucose. Cells were lysed by sonication and the fusion proteins were purified by Ni^2+^–NTA affinity chromatography. The purified proteins were dialyzed overnight in the presence of TEV protease, which cleaves the fusion proteins after the lipoyl domain. A second Ni^2+^–NTA affinity-chromatography step was carried out to remove the lipoyl domain. The domains were further purified by gel filtration using a HiLoad 26/60 Superdex 75 column (GE Healthcare). The elution volumes of both domains were consistent with their being monomeric. Double-deionized water was used to make the buffer solutions.

### 2.3. NMR spectroscopy   

Protein samples prepared for NMR spectroscopy experiments were typically at 1.5 m*M* in 90% H_2_O, 10% D_2_O containing 20 m*M* potassium phosphate pH 6.5, 100 m*M* NaCl, 5 m*M* β-mercaptoethanol. All spectra were acquired using a Bruker DRX800, DRX600 or DMX500 spectrometer equipped with pulsed field gradient triple resonance at 25°C, and were referenced relative to external sodium 2,2-dimethyl-2-silapentane-5-sulfonate (DSS) for proton and carbon signals or liquid ammonia for those of nitrogen. Assignments were obtained using standard NMR methods using ^13^C/^15^N-labelled, ^15^N-labelled, 10%^13^C-labelled and unlabelled protein samples (Bax *et al.*, 1991 ▸; Englander & Wand, 1987 ▸). Backbone assignments were obtained using the following standard set of two-dimensional and three-dimensional heteronuclear spectra: ^1^H–^15^N HSQC, HNCACB, CBCA(CO)NH, HNCACO, HNCO, HBHA(CO)NH and ^1^H–^13^C HSQC. Additional assignments were made using two-dimensional TOCSY and DQF–COSY spectra. Distance constraints were derived from two-dimensional NOESY spectra recorded with a mixing time of 120 ms. Torsional angle constraints were obtained from an analysis of C′, N, C^α^, H^α^ and C^β^ chemical shifts using *TALOS* (Cornilescu *et al.*, 1999 ▸). The stereospecific assignments of H^β^ resonances determined from DQF–COSY and HNHB spectra were confirmed by analyzing the initial ensemble of structures. Stereospecific assignments of H^γ^ and H^δ^ resonances of Val and Leu residues, respectively, were assigned using a fractionally ^13^C-labelled protein sample (Neri *et al.*, 1989 ▸). Once all NOEs had been assigned and initial structures had been calculated, hydrogen-bond constraints were included for a number of backbone amide protons for which signals were still detected after 10 min in a two-dimensional ^1^H–^15^N HSQC spectrum recorded in D_2_O at 278 K. Candidates for the acceptors were identified using *HBPLUS* for the hydrogen-bond donors that were identified by the H–D exchange experiments. When two or more candidates for acceptors were found for the same donor in different structures, the most frequently occurring candidate was selected. For hydrogen-bond partners, two distance constraints were used, where the distance ^(D)^H—O^(A)^ corresponded to 1.5–2.5 Å and ^(D)^N—O^(A)^ to 2.5–3.5 Å. The three-dimensional structures of the domains were calculated using the standard torsion-angle dynamics simulated-annealing protocol in *CNS* v.1.2 (Brunger, 2007 ▸). Residual dipolar couplings were measured for proteins aligned in 5% C12E5/1-hexanol. Alignment tensor values for the RDC constraints of the C-terminal domain were calculated using *SSIA* and the RDC constraints were incorporated in the final round of structure calculations. Structures were accepted where no distance violation was greater than 0.25 Å and where no dihedral angle violations were greater than 5°. The final coordinates have been deposited in the Protein Data Bank (PDB entries 4uzw and 4uzx).

To monitor the interaction of the SAP domain with DNA and RNA, ^15^N HSQC spectra of 200 µ*M* Tho1 SAP domain were recorded in the presence of 200 µ*M* self-complementary oligonucleotides corresponding to either a typical histone cluster scaffold attachment region sequence (5′-AGAAAATAATAAAATAAAACTAGCTATTTTATATTTTTTC-3′) or a random dsDNA sequence (5′-TCCTGATCAGGA-3′). The potential interaction with dsRNA was also measured using the 30-mer dsRNA oligonucleotide 5′-GGACAGCUGUCCCU­UCGGGGACAGCUGUCC-3′. The potential inter­action of the C-terminal domain with RNA was measured using ^15^N HSQC spectra recorded for 200 µ*M* C-terminal Tho1 domain in the absence and presence of several 200 µ*M* RNA and DNA oligonucleotides including 18-mer polyA, 18-mer polyU, 18-mer polyG, 18-mer polyC, the 30-mer dsRNA 5′-GGA­CAGCUGUCCCUUCGGGGACAGCUG­UCC-3′ and the 20-mer ssRNA 5′-CUUGUACAUAGU­UGGCCAUA-3′.

## 3. Results and discussion   

### 3.1. Cloning and domain-boundary selection   

**Figure 1:**
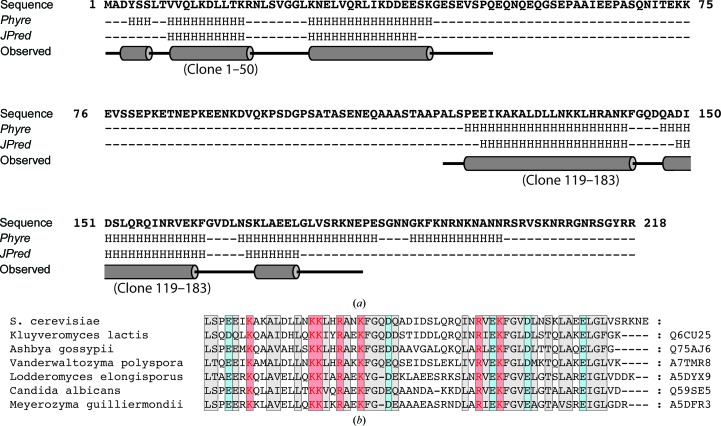
(*a*) Structure predictions for *S. cerevisiae* Tho1 by *Phyre* and *JPred*. The solution structures of the SAP domains and C-terminal domains determined are shown below the predictions. (*b*) Sequence alignment of *S. cerevisiae* Tho1 compared with sequences from *Kluyveromyces lactis*, *Ashbya gossypii*, *Vanderwaltozyma polyspora*, *Lodderomyces elongisporus*, *Candida albicans* and *Meyerozyma guilliermondii*. Residues with high sequence similarity and identity are shown in closed boxes, with basic, acidic and aliphatic residues coloured blue, red and grey, respectively.

*JPred* and *Phyre* both predicted Tho1 to contains two α-helical clusters, with *Phyre* predicting an additional helical motif at the C-terminus (Fig. 1 ▸ *a*). Careful analysis of the sequence and disorder prediction suggested that the additional α-helix predicted by *Phyre* would be unlikely to form. A ^15^N HSQC spectrum of a clone comprising residues 51–218 showed no additional resonances in the regions expected for structured residues (Supplementary Fig. S1). Subsequently, a number of clones were created to investigate the structures of the domains.

### 3.2. NMR assignments and data deposition   

**Figure 2:**
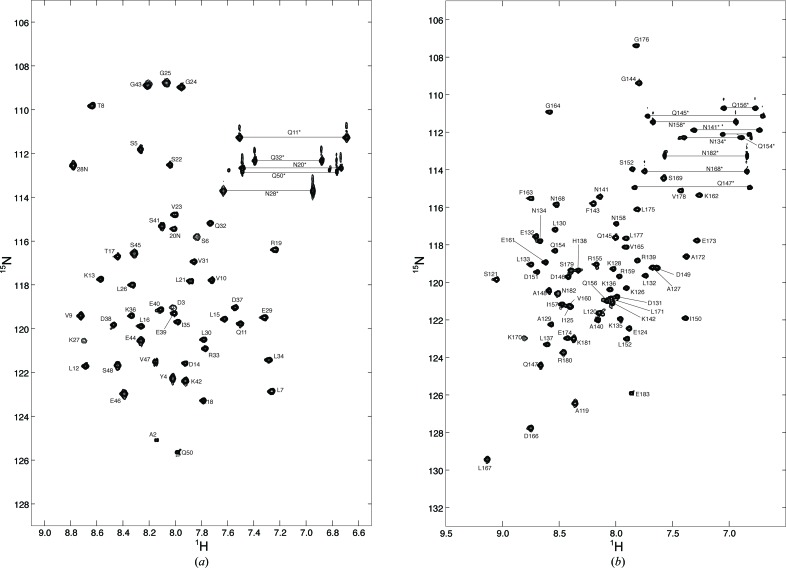
Two-dimensional ^1^H–^15^N HSQC spectra of the N-terminal SAP domain (*a*) and C-terminal domain (*b*) of *S. cerevisiae* Tho1 recorded at pH 6.5 and 293 K. The spectra were recorded on a Bruker DRX 500 MHz spectrometer with 1024 and 256 complex points along the *t*_2_ and *t*_1_ dimensions, respectively. The protein concentration was 1.5 m*M* in 95% H_2_O/5% D_2_O. The peaks are labelled with the single-letter amino-acid code followed by their respective sequence number, as established by sequence-specific assignments of the protein backbone.

Two clones comprising residues 1–50 and residues 119–183 of *S. cerevisiae* Tho1 were used for NMR assignment and structural analysis. We could assign 98% of the backbone resonances (only the N-terminal amide resonance and the amide N atoms of prolines were unassigned). All of the observable side-chain proton resonances were assigned using a combination of homonuclear and triple-resonance experiments as described in §[Sec sec2.3]2.3. The ^1^H_N_ and ^15^N resonance assignments for the proteins are shown by the single-letter code followed by the sequence number in the ^1^H–^15^N HSQC (Fig. 2 ▸).

### 3.3. Structural studies of the N-terminal SAP domain   

**Figure 3:**
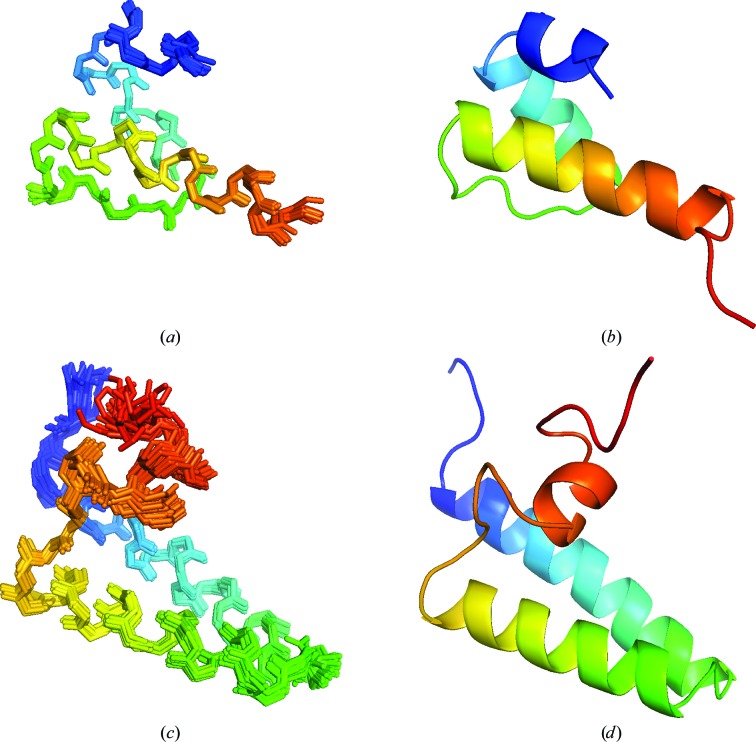
NMR structures of the yeast Tho1 domains. (*a*) Ensemble of 20 superimposed low-energy NMR-derived structures of the N-terminal SAP domain (backbone r.m.s.d. = 0.18 ± 0.08 Å) in ribbon representation. (*b*) Cartoon representation of the SAP domain. (*c*) Ensemble of 20 superimposed low-energy NMR-derived structures of the C-terminal domain (backbone r.m.s.d. = 0.47 ± 0.12 Å) in ribbon representation. (*d*) Cartoon representation of the C-terminal domain. Images were generated using *PyMOL*.

The structure of the SAP domain was determined using *CNS* v.1.2 from NOE, dihedral angle and hydrogen-bond restraints. Owing to the compact nature of the domain, residual dipolar couplings were not measured or used. A summary of all conformational constraints and statistics is presented in Table 1 ▸. The ensemble of structures calculated and a cartoon representation of the SAP domain are shown in Figs. 3 ▸(*a*) and 3 ▸(*b*), respectively. The SAP domain is composed of two α-helices (residues 9–19 and 27–42) connected by a structured loop in the helix–extended-loop–helix (HEH) motif typical of this fold. The N-terminus was structured from residue 2 onwards, whereas the C-terminal tail has few medium-range or long-range NOEs and was disordered. The structures of several SAP domains have been determined previously, with most having a role in DNA binding and chromosomal reorganization (Aravind & Koonin, 2000 ▸). Comparison with known structures using *DALI* shows that the Tho1 SAP domain is most similar to the structures of the SAP domains of SARNP (PDB entry 2do1; RIKEN Structural Genomics/Proteomics Initiative, unpublished work) and HNRNPUL1 (PDB entry 1zrj; RIKEN Structural Genomics/Proteomics Initiative, unpublished work), a protein that is also involved in the nuclear export of mRNA. DNA-binding experiments have revealed that the SAP domain of *S. cerevisiae* Tho1 has the potential to bind DNA (Jacobsen, 2003 ▸), but not dsRNA (Supplementary Fig. S2). The binding of the SAP domain to a random dsDNA 12-mer was investigated (Supplementary Fig. S3) and it was shown to bind in a manner consistent with other SAP domains (Okubo *et al.*, 2004 ▸)

### 3.4. Structural studies of the C-terminal domain   

*CNS* v.1.2 was used to determine a high-resolution solution structure of the domain using NOE, dihedral angle, hydrogen-bond and residual dipolar coupling (RDC) constraints. A summary of all conformational constraints and statistics is presented in Table 1 ▸. The ensemble of structures calculated and a cartoon representation of the C-terminal domain are shown in Figs. 3 ▸(*c*) and 3 ▸(*d*), respectively. The domain is composed of two antiparallel α-helices (residues 122–141 and 147–162) connected by a structured loop. Each of the antiparallel helices has a hydrophobic face and these faces pack together. The fold is further stabilized by two leucine residues in the C-terminal helix that interact with hydrophobic residues at the N-terminal end of helix 1 and the C-terminal end of helix 2. The N-terminus was structured from residue 120 onwards, whilst the C-terminal tail contained a structured loop and a small α-helix (residues 170–175). The residues after Ser179 contained no medium-range or long-range NOEs. A structure-comparison search using *DALI* revealed a similarity (r.m.s.d. of 2.7 Å over 50 residues) between the helix–turn–helix motif formed by the first two helices and the fold of the WHEP RNA-binding domain, which is found in multiple copies in a number of higher eukaryotic aminoacyl-transfer RNA synthetases. The C-terminal region of Tho1 has been shown to bind RNA (Jimeno *et al.*, 2006 ▸), and whilst there are several conserved positively charged residues in the domain (Fig. 1 ▸ *b*), the domain expressed (residues 119–183) exhibited no potential to bind RNA (Supplementary Fig. S4). The domain may still have the potential to bind to RNA, but the exact nature and sequence of the RNA required for binding is unknown. Alternatively, the domain may require the contribution of additional residues of Tho1 that were not included in the expression constructs used for this study.

### 3.5. Homologues of Tho1   

**Figure 4:**
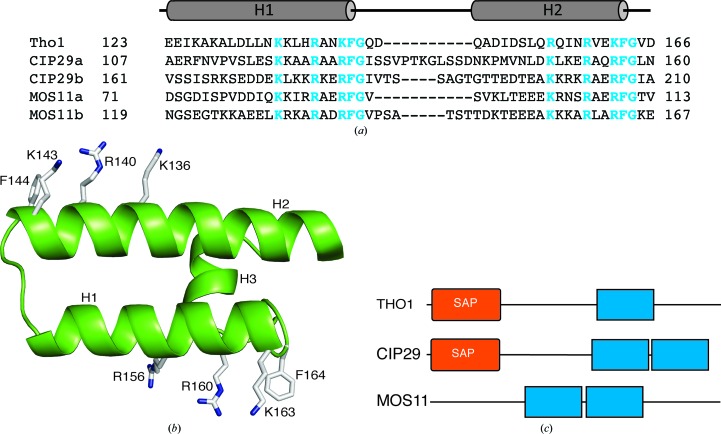
Sequence alignment of *S. cerevisiae* Tho1 compared with domains from human CIP29 and *Arabidopsis* MOS11. (*a*) Residues at the C-termini of the two helices with high sequence similarity and identity are shown in closed boxes and are coloured cyan. (*b*) The structure of the C-terminal domain showing the positions of the conserved residues. (*c*) Domain organization of *S. cerevisiae* Tho1 compared with human CIP29 and *Arabidopsis* MOS11.

A human protein, CIP29, has been proposed from sequence alignment to be a homologue of yeast Tho1 (Jimeno *et al.*, 2006 ▸). CIP29 contains a SAP domain, interacts with DNA, RNA and UAP56, and hence has been thought to have some role in transcription, RNA splicing, RNA export or translation (Aravind & Koonin, 2000 ▸; Hashii *et al.*, 2004 ▸; Leaw *et al.*, 2004 ▸; Sugiura *et al.*, 2007 ▸; Dufu *et al.*, 2010 ▸). CIP29 was initially reported to be a cytokine-induced protein and has been linked to several cancers (Choong *et al.*, 2001 ▸; Fukuda *et al.*, 2002 ▸; Hashii *et al.*, 2004 ▸; Leaw *et al.*, 2004 ▸), although the exact function of CIP29 is unknown. Comparison of the sequences of other members of the CIP29/Tho1 family (Fig. 4 ▸ *a*) reveals that the hydrophilic faces of the C-terminal ends of both of the helices in the helix–turn–helix motif are highly conserved. Each helix ends with a glycine residue, which is preceded by a phenylalanine that projects into solvent (Fig. 4 ▸ *b*). The residue preceding the phenylalanine and the residues one and two helical turns back from it are also highly conserved as either arginine or lysine. This produces two very similar potential RNA-binding sites at opposite ends of the domain that could, for example, interact with two copies of the same RNA sequence separated by a particular number of bases or specifically orientated within a structural motif. Inspection of the sequences of the C-terminal region of CIP29 shows that it contains a second closely spaced copy of this module, which can be readily identified by the presence of the lysine–arginine–phenylalanine–glycine sequence motif (Fig. 4 ▸ *c*). Two copies of this motif are also present in CIP29 homologues from other animal species. The *Arabidopsis* Tho1 homologue MOS11 (Germain *et al.*, 2010 ▸), together with homologous proteins from other plant species, also contains two copies of the motif but appears to lack an N-terminal SAP domain. Given the wide distribution of proteins containing two copies of the domain it is possible that the C-terminal copy has been lost in Tho1, with only the N-terminus of the first helix of the second domain being retained in the form of the small third helix, perhaps because it contributes to the stability of the fold. If this were the case, where both domains are present they would be expected to be orthogonal to each other. As well as binding to mRNA, all members of the Tho1/CIP29/MOS11 family characterized to date also bind to SUB2/UAP56 DEAD-box RNA helicases. As the C-terminal domain is the only strictly conserved region in this protein family, it may mediate these interactions as well.

## 4. Conclusions   

We report here the solution structures of the N-terminal SAP domain and C-terminal domain of yeast Tho1. The structures of the domains provide potential insight into the structure of related domains in the Tho1/CIP29/MOS11 family of proteins. The location of the DNA-binding site of the Tho1 SAP domain was shown to be similar to that observed in other SAP domains. The putative RNA binding of the C-terminal domain was investigated, although none was detected. Further work will be required to determine exactly which region of yeast Tho1 is responsible for RNA binding (Jimeno *et al.*, 2006 ▸). It is possible that RNA binding is mediated by a folding/binding event with a region of Tho1 that was not investigated in this study.

## Supplementary Material

PDB reference: *Saccharomyces cerevisiae* Tho1, SAP domain, 4uzw

PDB reference: C-terminal domain, 4uzx

Supporting Information: Supplementary Figures S1-S4.. DOI: 10.1107/S2053230X16007597/pq5029sup1.pdf

## Figures and Tables

**Table 1:** NMR and refinement statistics for the domains of *S. cerevisiae* Tho1

	SAP domain	C-terminal domain
NMR distance and dihedral constraints		
Distance constraints		
Total NOE	1260	1485
Intra-residue	469	600
Inter-residue	791	885
Sequential (|*i* − *j*| = 1)	255	342
Medium-range (|*i* − *j*| < 4)	294	360
Long-range (|*i* − *j*| > 5)	242	183
Residual dihedral coupling		45
Hydrogen bonds	52	64
Total dihedral angle restraints	97	123
φ	39	55
ψ	39	55
χ_1_	19	13
Structure statistics		
Violations (mean and s.d.)		
Distance constraints (Å)	0.0039 ± 0.0002	0.0114 ± 0.0018
Dihedral angle constraints (°)	0.121 ± 0.012	0.191 ± 0.029
Deviations from idealized geometry		
Bond lengths (Å)	0.0008 ± 0.00003	0.0020 ± 0.0002
Bond angles (°)	0.341± 0.002	0.402 ± 0.024
Impropers (°)	0.14 ± 0.005	0.31 ± 0.024
Average pairwise r.m.s.d.† (Å)	Residues 2–42	Residues 120–177
Heavy	0.64 ± 0.08	1.034 ± 0.06
Backbone	0.18 ± 0.08	0.47 ± 0.12

† Pairwise r.m.s.d. was calculated among the 20 lowest-energy structures without distance violations of >0.25 Å or dihedral angle violations of >5°.

## References

[bb1] Aguilera, A. (2005). *Curr. Opin. Cell Biol.* **17**, 242–250. 10.1016/j.ceb.2005.03.001 15901492

[bb2] Aravind, L. & Koonin, E. V. (2000). *Trends Biochem. Sci.* **25**, 112–114. 10.1016/s0968-0004(99)01537-6 10694879

[bb3] Bax, A., Ikura, M., Kay, L. E., Barbato, G. & Spera, S. (1991). *Ciba Found. Symp.* **161**, 108–119. 10.1002/9780470514146.ch8 1814691

[bb4] Brunger, A. T. (2007). *Nature Protoc.* **2**, 2728–2733. 10.1038/nprot.2007.406 18007608

[bb5] Cahuzac, B., Berthonneau, E., Birlirakis, N., Guittet, E. & Mirande, M. A. (2000). *EMBO J.* **19**, 445–452. 10.1093/emboj/19.3.445 PMC305581 10654942

[bb6] Choong, M. L., Tan, L. K., Lo, S. L., Ren, E.-C., Ou, K., Ong, S.-E., Liang, R. C. M. Y., Seow, T. K. & Chung, M. C. M. (2001). *FEBS Lett.* **496**, 109–116. 10.1016/s0014-5793(01)02409-7 11356193

[bb7] Cornilescu, G., Delaglio, F. & Bax, A. (1999). *J. Biomol. NMR*, **13**, 289–302. 10.1023/a:1008392405740 10212987

[bb8] Cuff, J. A., Clamp, M. E., Siddiqui, A. S., Finlay, M. & Barton, G. J. (1998). *Bioinformatics*, **14**, 892–893. 10.1093/bioinformatics/14.10.892 9927721

[bb11] Dufu, K., Livingstone, M. J., Seebacher, J., Gygi, S. P., Wilson, S. A. & Reed, R. (2010). *Genes Dev.* **24**, 2043–2053. 10.1101/gad.1898610 PMC2939366 20844015

[bb12] Englander, S. W. & Wand, A. J. (1987). *Biochemistry*, **26**, 5953–5958. 10.1021/bi00393a001 3689754

[bb13] Fukuda, S., Wu, D. W., Stark, K. & Pelus, L. M. (2002). *Biochem. Biophys. Res. Commun.* **292**, 593–600. 10.1006/bbrc.2002.6680 11922608

[bb14] Germain, H., Qu, N., Cheng, Y. T., Lee, E., Huang, Y., Dong, O. X., Gannon, P., Huang, S., Ding, P., Li, Y., Sack, F., Zhang, Y. & Li, X. (2010). *PLoS Genet.* **6**, e1001250. 10.1371/journal.pgen.1001250 PMC3009657 21203492

[bb15] Göhring, F., Schwab, B. L., Nicotera, P., Leist, M. & Fackelmayer, F. O. (1997). *EMBO J.* **16**, 7361–7371. 10.1093/emboj/16.24.7361 PMC1170336 9405365

[bb16] Hashii, Y., Kim, J. Y., Sawada, A., Tokimasa, S., Hiroyuki, F., Ohta, H., Makiko, K., Takihara, Y., Ozono, K. & Hara, J. (2004). *Leukemia*, **18**, 1546–1548. 10.1038/sj.leu.2403450 15284855

[bb17] Houseley, J., LaCava, J. & Tollervey, D. (2006). *Nature Rev. Mol. Cell Biol.* **7**, 529–539. 10.1038/nrm1964 16829983

[bb18] Huertas, P. & Aguilera, A. (2003). *Mol. Cell*, **12**, 711–721. 10.1016/j.molcel.2003.08.010 14527416

[bb19] Hurt, E., Luo, M. J., Röther, S., Reed, R. & Strässer, K. (2004). *Proc. Natl Acad. Sci. USA*, **101**, 1858–1862. 10.1073/pnas.0308663100 PMC357017 14769921

[bb20] Jacobsen, J. O. B. (2003). PhD thesis. Centre of Protein Engineering, Cambridge University.

[bb21] Jimeno, S., Luna, R., García-Rubio, M. & Aguilera, A. (2006). *Mol. Cell. Biol.* **26**, 4387–4398. 10.1128/MCB.00234-06 PMC1489133 16738307

[bb22] Jimeno, S., Rondón, A. G., Luna, R. & Aguilera, A. (2002). *EMBO J.* **21**, 3526–3535. 10.1093/emboj/cdf335 PMC126085 12093753

[bb23] Kelley, L. A. & Sternberg, M. J. (2009). *Nature Protoc.* **4**, 363–371. 10.1038/nprot.2009.2 19247286

[bb24] Köhler, A. & Hurt, E. (2007). *Nature Rev. Mol. Cell Biol.* **8**, 761–773. 10.1038/nrm2255 17786152

[bb25] Leaw, C. L., Ren, E. C. & Choong, M. L. (2004). *Cell. Mol. Life Sci.* **61**, 2264–2273. 10.1007/s00018-004-4205-x PMC11138947 15338056

[bb26] Meinel, D. M., Burkert-Kautzsch, C., Kieser, A., O’Duibhir, E., Siebert, M., Mayer, A., Cramer, P., Söding, J., Holstege, F. C. P. & Strässer, K. (2013). *PLoS Genet.* **9**, e1003914. 10.1371/journal.pgen.1003914 PMC3828145 24244187

[bb27] Neidhardt, F. C., Bloch, P. L. & Smith, D. F. (1974). *J. Bacteriol.* **119**, 736–747. 10.1128/jb.119.3.736-747.1974 PMC245675 4604283

[bb28] Neri, D., Szyperski, T., Otting, G., Senn, H. & Wüthrich, K. (1989). *Biochemistry*, **28**, 7510–7516. 10.1021/bi00445a003 2692701

[bb29] Okubo, S., Hara, F., Tsuchida, Y., Shimotakahara, S., Suzuki, S., Hatanaka, H., Yokoyama, S., Tanaka, H., Yasuda, H. & Shindo, H. (2004). *J. Biol. Chem.* **279**, 31455–31461. 10.1074/jbc.M403561200 15133049

[bb30] Piruat, J. I. & Aguilera, A. (1998). *EMBO J.* **17**, 4859–4872. 10.1093/emboj/17.16.4859 PMC1170815 9707445

[bb31] Poulsen, J. B., Sanderson, L. E., Agerschou, E. D., Dedic, E., Boesen, T. & Brodersen, D. E. (2014). *PLoS One*, **9**, e103470. 10.1371/journal.pone.0103470 PMC4111604 25062267

[bb32] Strässer, K., Masuda, S., Mason, P., Pfannstiel, J., Oppizzi, M., Rodriguez-Navarro, S., Rondón, A. G., Aguilera, A., Struhl, K., Reed, R. & Hurt, E. (2002). *Nature (London)*, **417**, 304–308. 10.1038/nature746 11979277

[bb33] Sugiura, T., Sakurai, K. & Nagano, Y. (2007). *Exp. Cell Res.* **313**, 782–790. 10.1016/j.yexcr.2006.11.014 17196963

[bb34] Zenklusen, D., Vinciguerra, P., Wyss, J.-C. & Stutz, F. (2002). *Mol. Cell. Biol.* **22**, 8241–8253. 10.1128/MCB.22.23.8241-8253.2002 PMC134069 12417727

