# Supplementary material for: High-resolution NMR structures of the domains of Saccharomyces cerevisiae Tho1
Source: Acta Crystallogr F Struct Biol Commun. 2016 May 23;72(Pt 6):500–6. doi: 10.1107/S2053230X16007597 (PMC4909252; doi:10.1107/S2053230X16007597)
Supplement: Supplementary file 1 [file f-72-00500-sup1.pdf]

STRUCTURAL BIOLOGY COMMUNICATIONS

Volume 72 (2016)

Supporting information for article:

High-resolution NMR structures of the domains of *Saccharomyces cerevisiae* Tho1

Julian O. B. Jacobsen, Mark D. Allen, Stefan M. V. Freund and Mark Bycroft

Figure S1

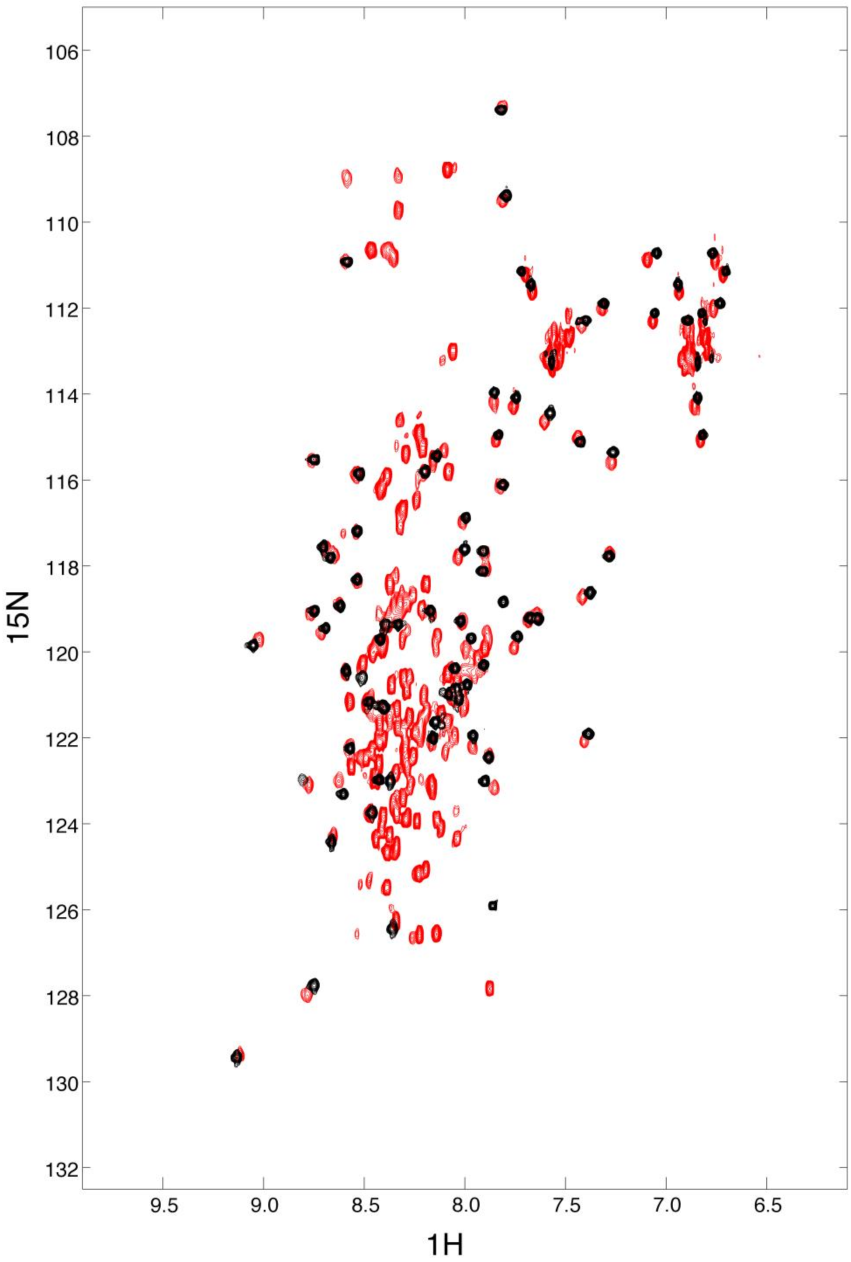

Superposition of 15N-HSQC spectra for tho1 (residues 119-183 - black) and tho1 (residues 51-218 - red) in PBS. The additional amide resonances in the spectrum for the larger domain appear to fall in the region typically associated with disordered residues.

Figure S2

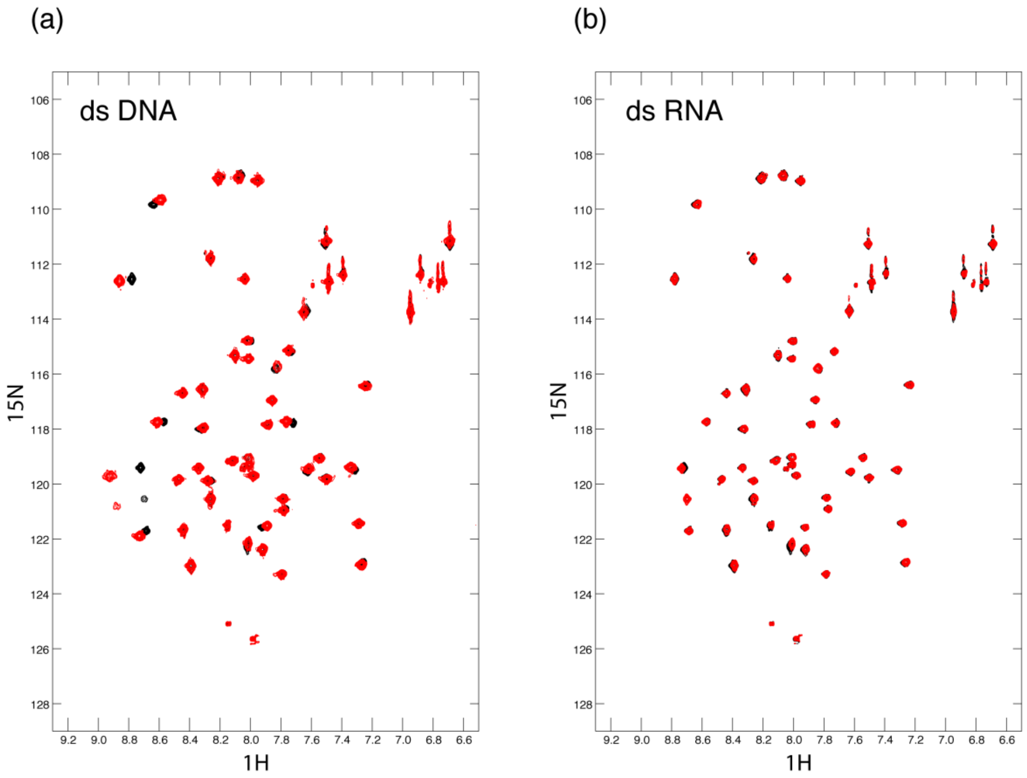

Superposition of 15N-HSQC spectra of Tho1 SAP domain (1-50) in PBS (black) with 15N-HSQC spectra of Tho1 SAP domain (1-50) in PBS with 200uM dsDNA and dsRNA (red). Whilst significant chemical shift perturbations were observed with dsDNA none were observed with dsRNA.

dsDNA 5′-TCCTGATCAGGA-3′

dsRNA 5′-GGACAGCUGUCCCUUCGGGGACAGCUGUCC-3′

Figure S3

(a)

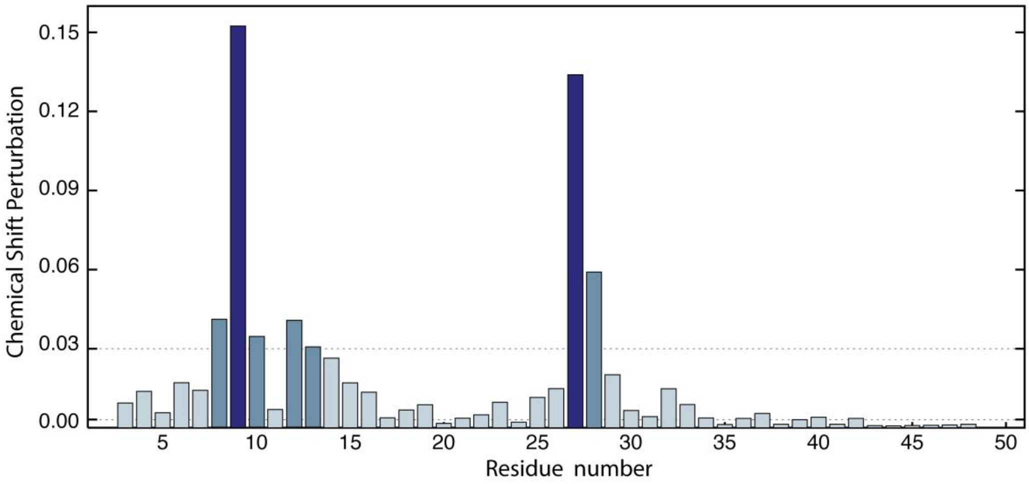

(b)

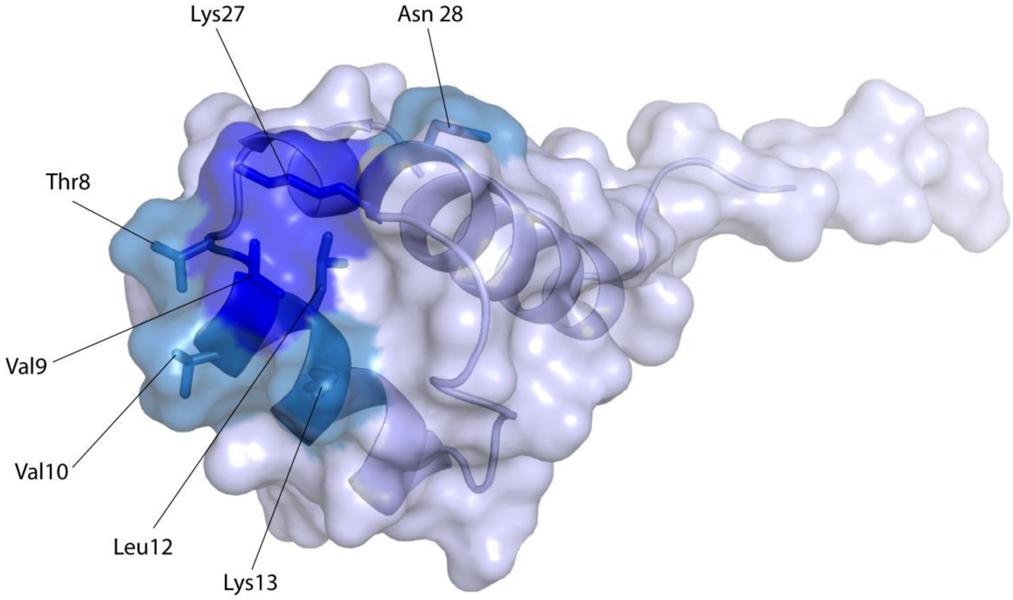

(a) CSP plot of *S. cerevisiae* Tho1 SAP domain on addtion of 200 μM 12-mer random dsDNA.

(b) CSP mapped on the structure of Tho1 SAP domain. Significant NCSPs were grouped and color-coded into two categories according to: medium (medium blue) if 2σ>CSP>1σ and strong (dark blue) if CSP>2σ, where σ is the standard deviation of the mean.

Figure S4

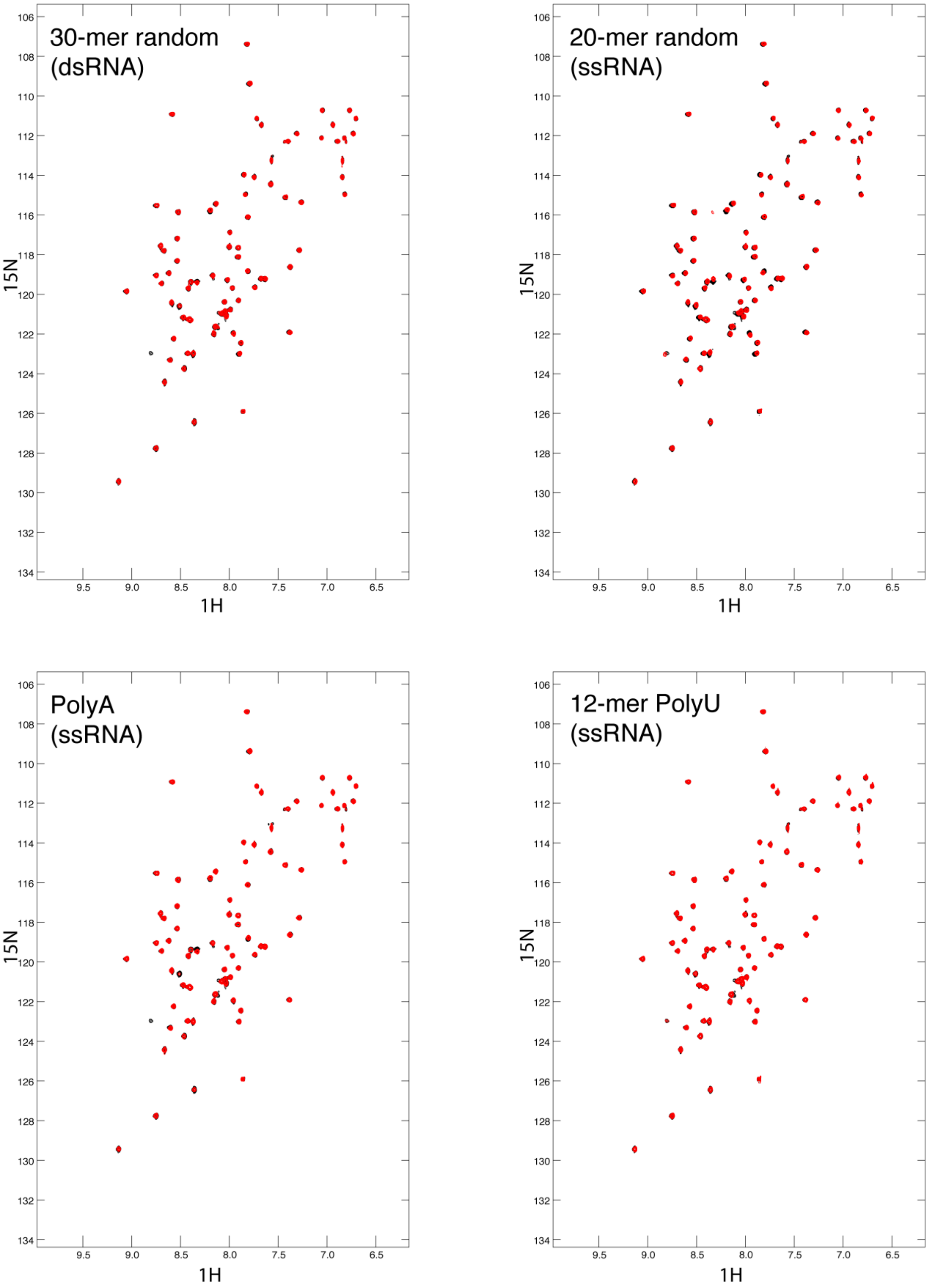

Superposition of 15N-HSQC spectra of tho1 (119-183) in PBS (black) with 15N-HSQC spectra of tho1 (118-183) in PBS with 200uM RNA (red).
No significant chemical shift perturbations were observed.

random 30-mer dsRNA 5’-GGACAGCUGUCCCUUCGGGGACAGCUGUCC-3
random 20-mer ssRNA 5’-CUUGUACAUAGUUGGCCAUA-3’
